# Posttraumatic stress, perceived hostile intention and reactions to peer provocation: A longitudinal study in US inner‐city youth

**DOI:** 10.1002/jcv2.70077

**Published:** 2025-12-04

**Authors:** Vladislav Ruchkin, Andrew Stickley, Mary Schwab‐Stone, Johan Isaksson

**Affiliations:** ^1^ Department of Medical Sciences Child and Adolescent Psychiatry Unit Uppsala University Uppsala Sweden; ^2^ Child Study Center Yale School of Medicine New Haven Connecticut USA; ^3^ Sala Forensic Psychiatric Clinic Sala Sweden; ^4^ Department of Preventive Intervention for Psychiatric Disorders National Institute of Mental Health National Center of Neurology and Psychiatry Kodaira Tokyo Japan; ^5^ Department of Women's and Children's Health Center of Neurodevelopmental Disorders (KIND) Centre for Psychiatry Research Karolinska Institutet & Stockholm Health Care Services Stockholm Sweden

**Keywords:** adolescents, aggression, gender, peer relationships, posttraumatic stress

## Abstract

**Background:**

Previous studies indicated that perceived hostile intention may mediate the link between traumatic experiences and aggressive behaviors, but the longitudinal associations between posttraumatic stress (PTS) and reactions to perceived provocation in adolescents have been underexplored. This study examined the longitudinal impact of PTS on the responses to and attribution of intentionality in peer provocation.

**Methods:**

The study was conducted with a representative sample of predominantly ethnic minority youth (*N* = 2014; 50.5% female; age 12–14 years old (*M* (SD) = 12.19 (0.83)); 60.6% African‐American, 27.6% Hispanic American, 10.1% White). The participants completed self‐reports on PTS in year 1 and appraised responses to hypothetical peer provocation scenarios that adolescents can encounter in their daily lives in year 2.

**Results:**

Higher levels of PTS were associated with more frequent choices of physical and verbal aggression, retaliation, teacher mediation, a less frequent choice of ignoring the situation and higher perceived hostile intention behind provocation. Compared to their female counterparts, boys more frequently chose reactions of physical aggression and retaliation, while girls more frequently reported verbal aggression and negotiation strategies. An interaction effect of PTS by gender suggested that compared to girls, boys with higher levels of PTS had more difficulty ignoring perceived peer provocation. Perceived hostile intention fully or partially mediated the association between PTS and physical aggression, verbal aggression, wish for retaliation, not being willing to negotiate, and the lower tendency to ignore provocation.

**Conclusion:**

High levels of PTS may significantly influence gender‐specific patterns of aggressive responses during conflicts with peers, while perceived hostile intention seems to mediate these associations. These findings may be relevant for the rehabilitation of traumatized youth, and should be taken into account when planning the treatment of adolescents with PTS.

## Key Points

**What's known?**

- Trauma exposure and posttraumatic stress (PTS) in adolescence are linked to elevated aggression and maladaptive conflict responses; hostile attribution biases (seeing others' intent as harmful) are established cognitive drivers of aggressive behavior.

**What's new?**

- In a longitudinal sample of urban, ethnic‐minority adolescents, higher PTS predicted more physical and verbal aggression and retaliation in response to peer provocation; perceived hostile intent fully mediated associations with aggression and partly mediated retaliation and avoidance; gender moderated the PTS‐ignoring link (stronger in boys).

**What's relevant?**

- Addressing hostile attribution bias and emotion regulation in trauma‐exposed youth, especially boys, may reduce reactive aggression and improve conflict resolution in school/community settings.

## Graphical abstract

This longitudinal study examined how posttraumatic stress (PTS) affects adolescents' responses to peer provocation, using self‐reports from 2014 predominantly ethnic minority youth aged 12–14. Path analysis showed that higher PTS was linked to more aggression, greater perceived hostility, and a lower tendency to ignore provocation, especially among boys. Perceived hostile intent mediated the associations between PTS and aggression, retaliation, low negotiation, and ignoring behavior.

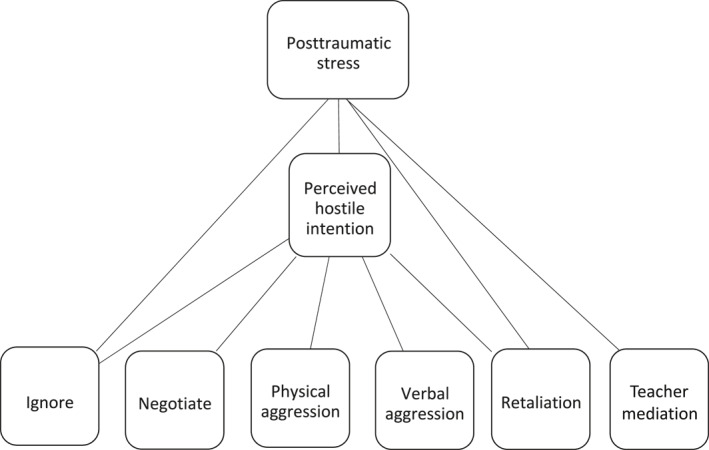

## INTRODUCTION

Youth violence continues to represent a serious public health challenge, with profound implications for both individuals and communities (Peterson et al., 2023). A better understanding of the factors underlying violent behavior, especially in adolescence, is essential for developing strategies to reduce the likelihood of violence and its adverse long‐term effects (David‐Ferdon et al., 2016). The relationship between trauma, specifically posttraumatic stress (PTS), and aggression has been well‐documented in the academic literature, yet many gaps remain, particularly in understanding the longitudinal processes by which trauma exposure shapes responses to interpersonal provocation (Margolin & Vickerman, 2007; Saunderson et al., 2023).

### Theoretical framework: social information processing model

The social information processing (SIP) model, which has traditionally focused on cognitive factors like information encoding, goal formation, and attribution of intent, offers a useful theoretical framework for understanding aggressive behavior. The model posits that adolescents interpret social cues based on their cognitive evaluations and these interpretations influence the emotional and behavioral responses to those cues (Crick & Dodge, 1994). While the SIP model has traditionally emphasized the role of cognitive processes, such as cue interpretation and intention attribution (Dodge & Crick, 1990), researchers have since highlighted the integral role of emotional and relational factors, such as emotional dysregulation, emotional intensity and duration, and interpersonal sensitivity in shaping how social information is processed (Arsenio & Lemerise, 2004; Kawabata & Ohbuchi, 2019; Lemerise & Arsenio, 2000). Emotional dysregulation and heightened emotional reactivity can bias adolescents toward interpreting ambiguous cues as hostile (Shields & Cicchetti, 1998), while individuals with high interpersonal sensitivity tend to perceive ambiguous or neutral social cues as threatening or rejecting (Downey & Feldman, 1996). These affective tendencies interact with cognitive processes, influencing intention attribution and response selection, and may significantly increase the likelihood of aggressive outcomes, particularly in emotionally charged or provocative situations (Arsenio & Lemerise, 2004).

### Perceived intentionality and aggression

The perceived intentionality of interpersonal provocation is a frequent precursor of aggression and the individual perception of being exposed to provocation tends to reduce self‐control and to increase feelings of anger and the likelihood and severity of aggression (Anderson & Bushman, 2002; Denson et al., 2011). Aggressive behavioral responses are hence influenced by an individual's tendency to perceive the intent of others as hostile, even when social context cues may be ambiguous (Milich & Dodge, 1984). At the same time, individual perceptions of events are processed based upon the encoding of prior and current experiences, knowledge, and context, which are then interpreted to form specific behavioral responses (Crick & Dodge, 1994; Firth, 2008).

### Impact of post‐traumatic stress on conflict responses

Previous experiences of severe traumatic stress may profoundly change the way people interpret and react to situations (Amstadter & Vernon, 2008), including those involving interpersonal provocation (Saunderson et al., 2023). More specifically, PTS is associated with both cognitive and affective alterations that contribute to increased aggression. Cognitively, PTS can result in hostile attribution biases, where ambiguous or neutral cues are interpreted as threatening or rejecting, increasing the likelihood of aggressive responses (Badour & Feldner, 2013; Dodge & Crick, 1990). Affectively, PTS is linked with heightened emotional reactivity, emotional dysregulation, and persistent negative emotional states such as anger, fear, or irritability, which can reduce the capacity to modulate impulses in emotionally charged situations (Marsee & Frick, 2010; Shields & Cicchetti, 1998). Neurobiologically, these changes are associated with hyperactivation of threat‐related brain circuits (e.g., the amygdala) and reduced prefrontal regulation, which together impair self‐control and increase reactive aggression (Kredlow et al., 2022). These vulnerabilities may become particularly apparent in the context of interpersonal provocation where a perceived threat may trigger specific cognitive attributions (Couette et al., 2020) and lead to dysregulated responses, even in the absence of actual danger (Badour & Feldner, 2013). As a result, adolescents with PTS may be especially prone to misinterpreting peer behavior as hostile and responding with aggression, due to disrupted cognitive appraisals and impaired emotion regulation capacities.

### Conflict resolution strategies and behavioral responses

Previous research has identified three primary types of response to interpersonal conflict: withdrawal, prosocial behavior, and aggression (e.g., Lindeman et al., 1997). In line with this, three distinct conflict resolution strategies have been proposed for adolescents (Fernet et al., 2016; Shulman et al., 2006). An avoidant (downplaying/withdrawing) strategy is characterized by efforts to minimize conflict exposure or to disengage physically or emotionally from the conflict situation without direct confrontation. It involves intentionally refraining from interaction with the provocateur or deflecting the issue to prevent escalation. An integrative (prosocial negotiation) strategy reflects constructive efforts to resolve differences through communication, compromise, or seeking mutual understanding. Finally, a conflictive (confrontational/aggressive behavior) strategy involves overtly confrontational responses aimed at imposing one's own will, including physical or verbal aggression. Additionally, given the frequent power imbalances in adolescent conflicts, two further behavioral strategies have been identified. Retaliation is operationally defined as a deliberate intent to “get even” or respond in kind to a previous provocation, often as a delayed or calculated response, rather than immediate avoidance or disengagement (Nishina & Juvonen, 2005; Recchia et al., 2019). Retaliation typically involves active efforts to impose harm or punishment on the provocateur, differentiating it from avoidance by its purposeful and reactive nature. Third‐party mediation involves the enlistment of an adult or authority figure, such as a teacher, to intervene in the conflict, which may serve dual roles, either facilitating prosocial negotiation or, alternatively, acting as a channel for seeking retribution through formal reprimand (e.g., Korpela et al., 2022; Shnabel & Nadler, 2010).

The choice of conflict resolution strategy, and in particular, the use of confrontational or aggressive responses, has been linked to the subjective perception of hostile intent during conflict (Martinelli et al., 2018). In relation to this, given the cognitive and emotional processing changes often associated with PTS, it is plausible that adolescents with PTS would exhibit differences in conflict responses, and specifically, a greater tendency to perceive hostile intent, an increased likelihood of aggressive reactions, reduced negotiation, and a stronger wish to retaliate.

### Gender differences in trauma and conflict responses

Previous research further suggests that responses both to traumatic experiences and to conflict may differ by gender. More specifically, as a reaction to stress, females seem more likely to exhibit emotional responses such as fear, anxiety, and helplessness (Chaplin et al., 2008; Lilly et al., 2009) and to experience more psychological problems (Axinn et al., 2013), including PTS (Alisic et al., 2014), whereas males tend to respond with higher levels of anger and less fear (Chaplin et al., 2008). Adolescent girls in general tend to score higher in communication skills (Black, 2000), react in a more prosocial/negotiating way (Eisenberg et al., 1991), and use constructive conflict resolution strategies more often than boys (Osterman et al., 1997). Thus, when it comes to situations of provocation and conflict, both adult and adolescent males tend to more often ascribe hostile intentions as being behind the conflict (Martinelli et al., 2018), to want to retaliate (Feld & Felson, 2008), to be more verbally and physically aggressive and to use more control strategies, whereas females may be more likely to avoid direct aggression and retaliation (Feld & Felson, 2008) and to use social aggression to harm a target (e.g., Feldman & Gowen, 1998; Heilbron & Prinstein, 2008).

### Study rationale and hypotheses

Research has demonstrated that PTS may lead to alterations in individual reactions to conflict situations and is associated with greater perceived hostile intentionality behind the conflict. Although previous studies have indicated that perceived hostile intention may mediate the link between traumatic experiences and aggressive behaviors (Van Voorhees et al., 2016; Zhu et al., 2020), the longitudinal impact of PTS on the way adolescents may react to peer provocation, as well as the potentially mediating role of perceived hostile intentionality have been underexplored (Griffith et al., 2025).

This study was conducted on a representative sample of predominantly ethnic minority youth due to their increased exposure to a range of contextual stressors, including community violence, socioeconomic disadvantage, and systemic discrimination, that place them at elevated risk for both trauma exposure and subsequent PTS symptoms (Andrews et al., 2015; Lopez et al., 2017; Slopen et al., 2016). Despite this heightened vulnerability, ethnic minority adolescents have been historically underrepresented in research on trauma and aggression. By focusing on this population, the study aimed to address this gap and ensure that findings are both relevant to and reflective of the lived experiences of youth who may be disproportionately affected by these challenges.

This study makes several novel contributions by expanding the SIP model to trauma‐exposed adolescents. First, it investigates the longitudinal relationship between PTS and aggressive responses to peer provocation, focusing on how trauma‐related factors shape social interactions over time. Second, it uniquely examines gender differences in how trauma exposure relates to aggression and conflict processing. Lastly, it highlights the sustained impact of PTS on adolescents' conflict resolution strategies and suggests potential causal pathways between trauma, cognitive processing, and conflict resolution.

We hypothesize that higher levels of PTS will correlate with more confrontational strategies (e.g., physical aggression, retaliation) and a greater tendency to perceive hostile intentions behind peer provocation. Furthermore, perceived hostile intention may mediate these relationships and gender will influence how PTS affects these reactions. The findings of this study will contribute to understanding trauma's role in shaping adolescent conflict resolution and highlight potential intervention targets for trauma‐exposed youth.

## MATERIALS AND METHODS

### Participants

A survey was conducted with all eligible students in the New Haven (CT) public school system, including students in alternative programs and bilingual classes (17 public middle and high schools). In the spring of year 1, the survey was completed by 2560 students in grades 6 and 7. This sample was then followed until the next survey administration 1 year later. Eighty percent of the original sample (*N* = 2056) completed the survey in year 2. High rates of attrition are common in longitudinal studies of urban ethnic minority adolescents (e.g., Patel et al., 2003), and related to high rates of school drop‐out and the high residential mobility of families, often driven by financial instability, job changes, or the need to find more affordable housing, which can disrupt children's education, create instability in social networks, and negatively impact overall well‐being. In addition, 42 (2.09%) adolescents had missing data on the outcome variables and were excluded from the analyses. No categorical background variables lacked data, as information on these variables was retrieved from the school register.

Analyses showed that the 546 students who dropped out or had missing data (compared to the 2014 remaining students) were older (*M* (SD) = 12.44 (1.00) versus 12.19 (0.83), *t* = 5.37, *p* < 0.001), more likely to be male (317 (58.1%) versus 996 (49.5%), *χ*2 = 12.73, *p* < 0.001), more likely to be White (72 (13.2%) versus 203 (10.1%) and less likely to be African‐American (306 (56.0%) versus 1220 (60.6%), *χ*2 = 8.42, *p* = 0.04), or to have a free/reduced lunch (407 (79.4%) versus 1662 (82.6%), *χ*2 = 32.48, *p* < 0.001). These drop‐out students did not differ from the follow‐up group in their PTS levels (*t* = 1.71, *p* = 0.87). These results suggest that attrition was selective across several key variables.

The final sample (*N* = 2014; 50.5% female; age at baseline 12–14 years (*M* (SD) = 12.19 (0.83)) was predominantly composed of minority ethnicities (60.6% African‐American, 27.6% Hispanic American, 10.1% White, and 1.7% other ethnicity), an accurate reflection of the local public school population (New Haven Public Schools, 2019). In the U.S., government‐supported programs allow reduced lunch fees or free lunches to be provided by schools in order to assist families with lower income levels. In connection with this, the sample population was predominantly socioeconomically disadvantaged, as reflected by the large proportion (over 82.6%), who qualified for free/reduced lunch status at either point of the data collection. Information about the students' eligibility for subsidized school lunches was obtained from the school register. This information was then used as a proxy for socioeconomic status (SES), with higher scores indicating worse SES (see below). Over 80 percent of the students' caregivers had the equivalent of a high school education or beyond.

### Survey procedure and ethical considerations

Parents were informed about the survey during school registration and received a follow‐up letter 2 weeks prior to its administration, which provided additional information and the opportunity to decline their child's participation. This passive informed consent procedure was approved by the university's institutional review board and was considered an ethically appropriate approach by the state legislature. Prior to the survey administration, students were presented with a detailed assent form that explained the nature of their participation and assured them of confidentiality. Students were asked to sign the form to indicate their assent. Rates of parent and student refusal were low, with less than 1% opting out. Students completed the survey in their classrooms during a single class period on a regular school day. Trained administrators conducted the session, reading each question aloud while students followed along in their individual booklets, reading silently and circling their responses. A second administrator was present in each classroom to provide individual assistance as needed. The survey was administered in both English and Spanish, allowing students to choose their preferred language. All students in the relevant grades who were present at school on the day of the survey administration were eligible to participate. For students who were absent, make‐up sessions were conducted at each school within 1 month of the initial administration.

### Measures

The *Child Post‐Traumatic Stress Reaction Index* (CPTS‐RI) is a 20‐item scale designed to assess PTS symptoms in school‐aged children and adolescents after exposure to a broad range of traumatic events (Steinberg et al., 2004). The frequency of symptoms is assessed on a Likert‐type five‐point rating scale ranging from “never” (0) to “most of the time” (4), where the total score can range from 0 to 80. The scale is internationally recognized and has well‐established cross‐cultural clinical cut‐offs based on the raw score, with higher scores on this instrument correlating closely with the DSM diagnosis of PTSD (Steinberg et al., 2004). Cronbach's alpha for the scale was 0.86.

We used a proxy variable for *SES*. Specifically, eligibility for free (scored 2) or reduced lunch (1) in year 1 was used as an index of SES and could hence vary between 0 and 2 with higher scores indicating lower SES. Students were eligible for this assistance if their family's income was less than 185% of the federal poverty threshold. The use of student eligibility for subsidized school lunches as a proxy measure of SES is in line with previous studies (e.g., Isaksson et al., 2024; Ruchkin et al., 2023).

The *Reaction to Peer Provocation (RPP) questionnaire* (Saunderson et al., 2023) was developed specifically for the purposes of the present study. It consists of 12 brief scenarios depicting hypothetical peer provocations that adolescents might encounter in school or community settings. These provocations were distributed across three categories: four scenarios involving social aggression (e.g., “You learned that another student was spreading mean rumors about you”), four describing verbal provocation (e.g., “A classmate made fun of you in front of your friends”), and four depicting physical aggression (“You had an argument with a classmate, and s/he shoved you”, or “A classmate bumped you from behind and you almost fell down”). After each scenario the respondent was asked: “What would you do?” (i.e., to indicate the type of reaction they would most likely have). They were prompted to select one of six response categories: “Ignore it” (=avoidance), “Wait and get even later” (=retaliation), “Discuss it together and try to solve this problem” (=negotiation), “Ask a teacher or another adult for help” (=teacher mediation), “Yell, curse or call the person names” (=verbal aggression), and “Push, hit or kick the person” (=physical aggression). Separate sum scores were calculated for each of these six response types (avoidance, retaliation, negotiation, teacher mediation, verbal aggression, and physical aggression) across all 12 scenarios (scored as 0 or 1 for each scenario), and hence potentially ranging from 0 to 12 for each of the six responses. Following each scenario, participants were also asked to evaluate the perceived hostile intention of the provocateur through the question: “The behavior of the classmate was…,” with four possible responses: “Definitely on purpose (=3),” “Possibly on purpose (=2),” “Possibly an accident (=1),” “Definitely an accident (=0)”. A total score for perceived hostile intention was calculated by summing responses across all 12 scenarios, yielding a possible range from 0 to 36, with higher scores indicating a greater perceived hostile intention. Details of the instrument are provided in Supporting Information S1: Appendix S1.

### Statistical analyses

Data were analyzed using SPSS (version 28). Independent sample *t*‐tests were used for univariate comparisons of the study variables across gender. A full path analysis (R Studio Version 2024.04.2; lavaan package) using robust maximum likelihood estimation was performed to assess how reactions to perceived peer provocation (i.e., by physical aggression, verbal aggression, negotiation, asking the teacher, avoidance, retaliation), as well as perceived hostile intention, were associated with PTS 1 year earlier, also adjusting for gender, age and SES. In the path analysis, the intercorrelations of the independent and dependent variables were adjusted for. We also investigated any interaction effect of gender by PTS on the reaction to perceived peer provocation and perceived hostile intention in a separate model, also adjusting for age and SES. Lastly, we included the perceived hostile intention of the provocateur as a mediating variable in the full path analyses in order to assess the indirect effect of this variable on the association between PTS and each choice of reaction to perceived peer provocation. Potential interaction effects by gender were not investigated in this last model. In the analyses, two‐tailed tests with a *p*‐value <0.05 were considered statistically significant.

## RESULTS

### Prevalence of PTS and the RPP by gender

As shown in Table 1, girls reported higher levels of PTS, as well as more verbal aggression and negotiation in relation to the scenarios compared to boys, whereas boys more often chose to react with physical aggression and retaliation, compared to girls. Overall, the frequency of perceived hostile intention was high across the participants with a mean value of 2.74 for each scenario and did not differ by gender.

**TABLE 1:** Study variables *M* (SD) by gender.

	All (*N* = 2014)	Females (*n* = 1018)	Males (*n* = 996)	*t*‐test
Physical aggression	1.49 (2.05)	1.32 (1.82)	1.67 (2.25)	3.77***
Verbal aggression	1.54 (2.09)	1.71 (2.18)	1.37 (1.96)	3.64***
Negotiation	1.58 (2.13)	1.91 (2.32)	1.25 (1.86)	7.01***
Teacher mediation	0.32 (0.97)	0.32 (0.91)	0.32 (1.02)	0.12
Ignore	4.65 (3.43)	4.66 (3.33)	4.63 (3.54)	0.19
Retaliation	1.82 (2.14)	1.56 (1.94)	2.09 (2.92)	5.62***
Hostile intention	32.93 (8.88)	33.00 (8.26)	32.85 (9.48)	0.36
PTS	22.47 (13.57)	24.17 (14.13)	20.73 (12.75)	5.74***
Age	12.19 (0.83)	12.14 (0.80)	12.25 (0.85)	3.00**
SES	1.51 (0.77)	1.54 (0.76)	1.48 (0.79)	1.72*

Abbreviations: *M*, mean; PTS, posttraumatic stress; SD, standard deviation; SES, socio‐economic status.

*p < .05, **p < .01, ***p < .001.

### Associations between PTS and the RPP

The path analytic model is presented in Table 2. In the full model, only a small proportion of the variance in the reactions to perceived peer provocation (1%–3%) was explained in the model. Higher levels of PTS were associated with more frequent choices of physical and verbal aggression, retaliation, teacher mediation, a less frequent choice of ignoring the situation and more frequently ascribed hostile intention. Gender differences were also found, where boys more frequently chose reactions of physical aggression and retaliation, while girls more frequently reported verbal aggression and negotiation strategies. Increasing age was associated with a less frequent choice of negotiation and less frequently ascribed hostile intention, and higher SES was related to teacher mediation and a less frequent choice of retaliation. In a separate model, investigating a possible interaction by gender, the association between PTS and ignoring peer provocation was found to differ by gender (*β* = −0.092; *p* = 0.043), where a negative association between PTS and ignoring provocation was stronger among boys, indicating that boys with greater levels of PTS had more difficulty ignoring perceived peer provocation.

**TABLE 2:** Results of the full path analysis of participants' reactions to interpersonal provocation regressed on PTS and covariates, with standardized regression coefficients (*β*), also adjusting for the inter‐correlations of independent and dependent variables.

	Physical aggression	Verbal aggression	Negotiation	Teacher mediation	Ignore	Retaliation	Hostile intention
PTS	0.063**	0.059**	−0.032	0.062**	−0.096***	0.062**	0.094***
Gender (male)	0.093***	−0.075**	−0.154***	0.008	−0.017	0.130***	0.006
Age	−0.010	0.016	−0.060**	−0.015	0.024	−0.007	−0.052*
SES	0.008	−0.024	−0.001	0.054*	0.018	−0.067**	−0.023
*R* ^2^	0.011	0.011	0.028	0.007	0.010	0.023	0.012
PTS × gender^a^	0.006	−0.061	0.072	0.019	−0.092*	0.060	−0.006

Abbreviations: PTS, posttraumatic stress; SES, socio‐economic status.

^a^ Assessed in a separate model.

*p < .05, **p < .01, ***p < .001.

### Perceived hostile intention as a mediator between PTS and the RPP

**FIGURE 1:**
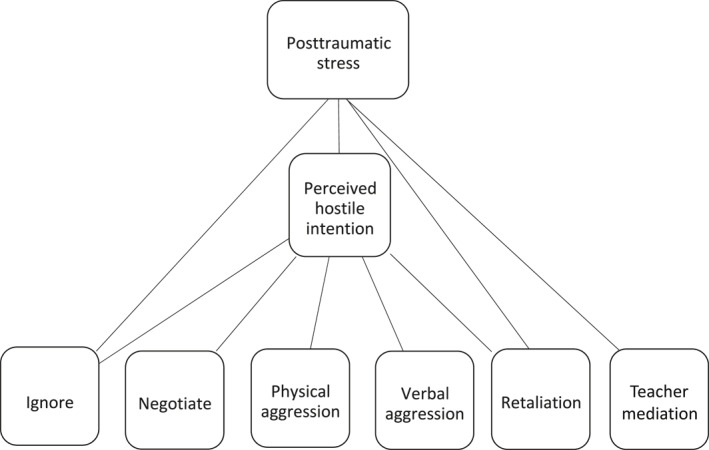
Longitudinal associations between posttraumatic stress and conflict resolution strategies mediated by perceived hostile intention.

The mediation analysis is presented in Table 3 and Figure 1. In the full model, a higher perceived hostile intention of the provocateur was shown to fully mediate the association between PTS and physical aggression, verbal aggression, and not being willing to negotiate. Accordingly, the explained variance of the outcomes increased, where the model explained 11% of physical aggression, 7% of verbal aggression and 4% of negotiation. Partial mediation by perceived hostile intention was also found for the associations between PTS and a lower tendency to ignore the provocation and a more frequent wish to retaliate.

**TABLE 3:** Mediation by perceived hostile intention of the relationships between PTS and the choice of reaction to interpersonal provocation.

Relationship	Total effect	Direct effect	Indirect effect	Confidence interval		*R* ^2^	Conclusion
				Lower	Upper		
PTS → hostile intention → physical aggression	0.062**	0.034	0.028***	0.014	0.042	0.105	Full mediation
PTS → hostile intention → verbal aggression	0.059**	0.036	0.022**	0.011	0.023	0.069	Full mediation
PTS → hostile intention → negotiation	−0.032	−0.022	−0.010**	−0.015	−0.004	0.040	Full mediation
PTS → hostile intention → teacher mediation	0.062**	0.064**	−0.003	0.000	−0.007	0.008	No mediation
PTS → hostile intention → ignore	−0.096***	−0.089***	−0.007*	−0.012	−0.001	0.015	Partial mediation
PTS → hostile intention → retaliation	0.062**	0.048*	0.013***	0.006	0.021	0.044	Partial mediation

Abbreviation: PTS, posttraumatic stress.

*p < .05, **p < .01, ***p < .001.

## DISCUSSION

Our findings were consistent with previous research (Margolin & Vickerman, 2007; Saunderson et al., 2023) and suggested that adolescents with greater levels of PTS may react differently in situations of interpersonal provocation, as compared to their peers. The relationship between PTS and reactions to peer provocation may be influenced by both gender and the perceived hostile intention behind the provocation.

### Aggression and conflict resolution in adolescents with PTS

Results suggest that adolescents with PTS tend to be less likely to ignore conflict and more likely to react with physical and verbal aggression, as well as with a wish to retaliate. This reaction pattern, described in traumatized individuals previously, suggests that some aspects of PTS may lead to maladaptive conflict resolution strategies, perpetuating aggressive behavior.

Several theoretical explanations have been advanced to explain the link between PTS and aggression. Individuals with PTS may perceive noxious stimuli as too intense and neutral stimuli as harmful, potentially leading to maladaptive coping strategies (Mariotti, 2015). PTS also impacts behavior and emotion regulation, influencing the ability to evaluate and modify affective experience and expression (Gratz & Roemer, 2004). Arousal in response to threat may interfere with higher level cognitive functioning, thereby weakening inhibition against aggression (Mohr et al., 2007). It has been further suggested that negative cognitions and affect in PTS may connect through associative networks with anger‐related feelings, thoughts, and aggressive inclinations (Taft et al., 2007). In addition, PTS may impair cognitive capacity to adequately identify risk and to exit harmful situations and hence, challenge self‐protective behavior (Orcutt et al., 2002). Some theories have thus suggested that traumatized individuals develop a cognitive bias related to misperception of threat in ambiguous contexts, leading to a threat‐anger program that facilitates aggression (Novaco & Chemtob, 1998). Whiting and Bryant (2007) for example, found a strong association between maladaptive appraisals and posttraumatic anger. Lastly, physiological changes resulting from PTS might make traumatic experiences “addictive”, further potentiating involvement in violence (e.g., Haapasalo & Pokela, 1999).

### Hostile attributional style and trauma

Research has identified several factors influencing the development of a hostile attributional style, which is enduring and mediates behavior, including a history of trauma, modeling of hostile attributions by adults and peers, and cultural values emphasizing self‐defense and retaliation (Denson et al., 2011). In relation to the above, PTS strongly influences cognitive‐emotional processes and affects interpretation of events, making it less likely for traumatized individuals to ignore provocation and more likely for them to respond aggressively and seek retaliation. Previous research has similarly shown that PTS significantly predicts physical aggression over time, with hostile attributions mediating this association, suggesting the potential utility of targeting hostile cognitions in therapy for anger and aggression (Van Voorhees et al., 2016).

### Retaliation and trauma

Retaliation is often seen as a major reason for conflict continuing beyond its original cause (e.g., Kim & Smith, 1993). Studies suggest that the desire for revenge is a part of children's normal experiences of conflict (Recchia et al., 2019). Decision‐making about retaliation develops prior to adolescence, involving both assessment of intentionality behind the conflict and preferred ways of retaliating (Bloom, 2001), but most youth recognize their capacity to contain and redirect these desires (Recchia et al., 2019). Research has further indicated that a wish for retaliation can arise from traumatic experiences, related to the feelings of injustice, harm, shame and humiliation (Orth et al., 2006). Previous studies (Vossekuil et al., 2002) found, for example, that many of the attackers in targeted school violence described retaliation as a primary cause of their violent behavior. Consistent with prior literature (e.g., Gäbler & Maercker, 2011; Van Voorhees et al., 2016), we found that higher levels of PTS, especially in males, were associated with a wish to retaliate, partially mediated by perceived hostile intent.

Considering cognitive‐emotional alterations related to trauma, it has been suggested that traumatized children might develop “malignant belief systems” (Stien & Kendall, 2004), where hopelessness, despair, and hatred drive future retaliatory violence (Haen & Weber, 2009), and where traumatized individuals may struggle to break a “repetitive cycle of shame‐attracting behavior followed by vengeful behavior” (Bloom, 2001, p. 10). Although revenge is intended to restore power and control, the punishment of perpetrators or retaliation rarely succeeds in bringing relief from trauma or compensate for harm (Herman, 1992; Orth, 2004). Hence, helping traumatized children to find more constructive conflict resolution strategies is an imperative.

### Alternative theoretical perspectives

While this study focused on threat perception, cognitive‐affective alterations, and hostile attribution as mechanisms linking PTS to conflict responses, other theoretical frameworks could further enrich understanding of these associations. Attachment theory suggests that early trauma and disruptions in caregiver relationships contribute to insecure attachment styles, which in turn are associated with difficulties in emotion regulation, interpersonal functioning, and increased vulnerability to aggression (Mikulincer & Shaver, 2007). Adolescents with disorganized or avoidant attachment may interpret peer interactions through a lens of distrust or threat, reinforcing maladaptive conflict strategies (Doyle & Cicchetti, 2017). Similarly, the general psychopathology (p‐factor) model proposes that trauma may contribute to broad vulnerability to psychopathology, encompassing externalizing and internalizing symptoms (Caspi et al., 2014; Lahey et al., 2012). From this perspective, the observed behavioral responses may reflect broader dysregulation rather than trauma‐specific effects alone. Including these alternative pathways in future research could help disentangle trauma‐specific from more general developmental risks and help identify tailored interventions.

### Teacher mediation in conflict resolution

Teacher mediation following peer provocation appears to be a potentially positive alternative for conflict resolution among traumatized individuals. Indeed, studies suggest that teacher‐led mediations may help students to actively resolve conflicts, express opinions, negotiate peer relationships and develop competence in social interactions and relationship management (Cekaite, 2020; Korpela et al., 2022). Having said this, the involvement of a third party may also serve as an opportunity to reprimand the other party for the damage caused (Shnabel & Nadler, 2010), possibly reflecting an attempt at retribution through the teacher. However, given the observed lack of mediation by perceived hostile intention in the association between PTS and teacher mediation, this suggests that this strategy may have had a more constructive and prosocial nature.

### Gender differences

Girls reported higher levels of PTS than boys, aligning with previous research indicating that while males may be more likely to be exposed to diverse traumatic events (e.g., Breslau et al., 1991), females are more likely to report PTS symptoms (e.g., Alisic et al., 2014; Garza & Jovanovic, 2017; Singer et al., 1995). In addition, girls generally respond more emotionally to undesirable life events (Kessler & McLeod, 1984), and from adolescence onwards have a greater risk of developing PTS compared to boys (Alisic et al., 2014).

Regarding reactions to hypothetical peer provocation by gender, girls were more likely to negotiate or react verbally, whereas boys were more likely to react with physical aggression and wish for retaliation. Gender differences in aggressive behavior may be culturally determined, discouraging aggression and favoring negotiation in females (Holliday et al., 2015). Indeed, socialization processes and contextual factors play a crucial role in shaping these gendered patterns of behavior, as societal expectations and norms often encourage boys to express aggression outwardly and girls to adopt more relational or verbal strategies, thus influencing how trauma responses are manifested. Boys' tendency for physical aggression may also reflect gender differences in information processing and response styles. Boys, for example, are more likely to externalize negative affect and to respond with anger/aggression, while girls internalize their responses (Chen et al., 2012). At the same time, negotiation and compromise, often seen as more consistent with female gender stereotypes, represent more constructive conflict resolution methods, linked to better well‐being and mental health (Feldman & Gowen, 1998) and should be encouraged in adolescents for conflict resolution.

Among traumatized individuals, boys with higher PTS had more difficulty ignoring peer provocation. These findings align with gender differences in trauma‐related aggression (Isaksson et al., 2020) and support previous research that suggests that boys may react to trauma with externalizing behaviors, rather than internalizing it (Gorman‐Smith & Tolan, 1998; Miller et al., 1999).

### Strengths and limitations

This study had a number of strengths, such as having a longitudinal design while using data from a large sample of predominantly ethnic minority inner‐city adolescents, where a variety of measures were used to evaluate the associations between PTS and reactions to peer provocation. However, it also had several limitations. First, as the assessment of PTS was based on self‐reports, reporting bias may have been an issue and the use of structured screening/clinical interviews would have been beneficial to complement the assessment. Second, the RPP questionnaire describes a set of hypothetical scenarios, which may not fully reflect actual behaviors in real‐life situations of peer provocation. Third, attrition led to the exclusion of 504 students who left school and 42 with incomplete data were also excluded from the study. Since we had a relatively large sample that could detect small effect sizes, a complete‐case analysis was used for dealing with missing data. However, this might have increased the risk of the data not being representative. At the same time, previous research has reported that loss to follow‐up rarely affects estimates of association (Saiepour et al., 2019; Wolke et al., 2009) and methods for adjusting for the loss of cases, such as multiple imputation, involve assumptions that the data are missing at random, which is why a more stringent approach was chosen. Fourth, the present study was limited to predominantly ethnic minority adolescents from an inner‐city environment, and the results may differ in other adolescent groups. Additionally, the study did not assess gender identities beyond the self‐reported binary male/female categorization, limiting understanding of how PTS and reactions to peer provocation may manifest in non‐binary or gender‐diverse adolescents, which represents an area for future research. Finally, while statistically significant, the effect sizes that represent the unique amount of variance explained by each predictor variable were small (Cohen, 1988) and hence our findings should be interpreted with caution.

## CONCLUSIONS

PTS seems to be important in determining the response to peer provocation. Results suggest that PTS may be correlated with more aggressive behavioral tendencies, as well as with hostile attributional styles, and indicate that some of the differences in the reactions to peer provocation in the wake of traumatic exposure may be attributable to gender‐specific mechanisms. This study further clarifies how interpersonal aggression can be shaped by traumatic experiences and supports the relevance of cognitive‐emotional constructs in the context of trauma. These findings may be relevant for the rehabilitation of traumatized individuals and should be taken into account when planning the treatment of adolescents with PTS. Our findings further highlight the importance of the gender‐specific aspects of the cognitive‐emotional processing of trauma, and may help explain the heterogeneity of gender‐related differences in conflict situations in connection with PTS.

## AUTHOR CONTRIBUTIONS

**Vladislav Ruchkin**: Conceptualization; data curation; formal analysis; methodology; project administration; writing—original draft. **Andrew Stickley**: Conceptualization; writing—original draft. **Mary Schwab‐Stone**: Conceptualization; investigation; methodology; project administration; writing—review and editing. **Johan Isaksson**: Data curation; formal analysis; writing—original draft. All authors read and approved the final manuscript.

## CONFLICT OF INTEREST STATEMENT

The authors declare no conflicts of interest.

## ETHICAL CONSIDERATIONS

Informed consent and parental assent have been appropriately obtained (HIC #7092 from 2003 to 09‐02, continuation of Project #6985 under the Yale University HIC Committee on Research Involving Human Subjects) and considered as an appropriate ethical procedure by the state legislature.

## Supporting information

Supporting Information S1

## Data Availability

The data are not publicly available due to the initial decision of the local ethics committees, as well as the restrictions included in the informed consent statement where it was stated that the data would only be used by the research group and would not, in other than aggregated form, be utilized elsewhere.
