# Supplementary material for: Posttraumatic stress, perceived hostile intention and reactions to peer provocation: A longitudinal study in US inner‐city youth
Source: JCPP Adv. 2025 Dec 4:e70077. Online ahead of print. doi: 10.1002/jcv2.70077 (PMC13339556; doi:10.1002/jcv2.70077)
Supplement: Supplementary file 1 — Supporting Information S1 [file JCV2-9999-e70077-s001.doc]

**Posttraumatic stress, perceived hostile intention and reactions to peer provocation:**

**A longitudinal study in US inner-city youth**

**Supporting Information**

**Appendix S1.** Details of the Instrument

**Imagine that each of the situations described below happens between you and another student of the same age and gender as you are. After each situation there are two questions. For the first question, choose the answer that is closest to *how you would act* in this situation. For the second question, circle the answer that best describes the *behavior of the other student*.**

| **1** | | **A classmate made fun of you in front of your friends.** | | | | | | | | | | | | | | | | | | | | | | | | | | | | | | | | | | |
| --- | --- | --- | --- | --- | --- | --- | --- | --- | --- | --- | --- | --- | --- | --- | --- | --- | --- | --- | --- | --- | --- | --- | --- | --- | --- | --- | --- | --- | --- | --- | --- | --- | --- | --- | --- | --- |
|  | | **a.** | | | | | | | What would you do (circle one)? | | | | | | | | | | | | | | | | | | |  | | | | | |  | | |
|  | | | | | | | | | **Ignore it** | | **Wait and get even later** | | | | | | | | **Discuss it together and try to solve the problem** | | | | | | | **Ask a teacher or other adult for help** | | | **Yell, curse, or call the person names** | | | | | | **Push, hit, or kick the person** | |
|  | | **b.** | | | | | | | The behavior of the classmate was… | | | | | | | | | | | | | | | | | | |  | | | | | |  | | |
|  | | |  | | | | | | **Definitely on purpose** | | | | | | | | **Possibly on purpose** | | | | | | | | **Possibly an accident** | | | | | | | **Definitely an accident** | | | | |
| **2** | | **A classmate is having a party, many of your friends are going, but no one told you about it.** | | | | | | | | | | | | | | | | | | | | | | | | | | | | | | | | | | |
|  | | **a.** | | | | | | | What would you do (circle one)? | | | | | | | | | | | | | | | | | | |  | | | | | |  | | |
|  | | | | | | | | | **Ignore it** | | **Wait and get even later** | | | | | | | | **Discuss it together and try to solve the problem** | | | | | | | **Ask a teacher or other adult for help** | | | **Yell, curse, or call the person names** | | | | | | **Push, hit, or kick the person** | |
|  | | **b.** | | | | | | | The behavior of the classmate was… | | | | | | | | | | | | | | | | | | |  | | | | | |  | | |
|  | |  | | | | | | | **Definitely on purpose** | | | | | | | | **Possibly on purpose** | | | | | | | | **Possibly an accident** | | | | | | | **Definitely an accident** | | | | |
| **3** | | **You and a classmate made a plan to go somewhere, but s/he didn’t show up.** | | | | | | | | | | | | | | | | | | | | | | | | | | | | | | | | | | |
|  | | **a.** | | | | | | | What would you do (circle one)? | | | | | | | | | | | | | | | | | | |  | | | | | |  | | |
|  | | | | | | | | | **Ignore it** | | **Wait and get even later** | | | | | | | | **Discuss it together and try to solve the problem** | | | | | | | **Ask a teacher or other adult for help** | | | **Yell, curse, or call the person names** | | | | | | **Push, hit, or kick the person** | |
|  | | **b.** | | | | | | | The behavior of the classmate was… | | | | | | | | | | | | | | | | | | |  | | | | | |  | | |
|  | |  | | | | | | | **Definitely on purpose** | | | | | | | | **Possibly on purpose** | | | | | | | | **Possibly an accident** | | | | | | | **Definitely an accident** | | | | |
| **4** | | **A classmate bumped you from behind and you almost fell down.** | | | | | | | | | | | | | | | | | | | | | | | | | | | | | | | | | | |
|  | | **a.** | | | What would you do (circle one)? | | | | | | | | | | | | | | | | | | | | | | |  | | | | | |  | | |
|  | | | | | **Ignore it** | | | | | | **Wait and get even later** | | | | | | | | **Discuss it together and try to solve the problem** | | | | | | | **Ask a teacher or other adult for help** | | | **Yell, curse, or call the person names** | | | | | | **Push, hit, or kick the person** | |
|  | | **b.** | | | The behavior of the classmate was… | | | | | | | | | | | | | | | | | | | | | | |  | | | | | |  | | |
|  |  | | | | **Definitely on purpose** | | | | | | | | | | **Possibly on purpose** | | | | | | | | **Possibly an accident** | | | | | | | **Definitely an accident** | | | | | | |
|  |  | | | |  | | | | | | | |  | | | | | | | |  | | | | | | | | | | |  | | | |  |
| **5** | **You had an argument with a classmate, and s/he shoved you.** | | | | | | | | | | | | | | | | | | | | | | | | | | | | | | | | | | | |
|  | **a.** | | | What would you do (circle one)? | | | | | | | | | | | | | | | | | | | | | | | |  | | | | | |  | | |
|  | | | | **Ignore it** | | | | | | | | **Wait and get even later** | | | | | | | | **Discuss it together and try to solve the problem** | | | | | | **Ask a teacher or other adult for help** | | | **Yell, curse, or call the person names** | | | | | | **Push, hit, or kick the person** | |
|  | **b.** | | | | The behavior of the classmate was… | | | | | | | | | | | | | | | | | | | | | | |  | | | | | |  | | |
|  |  | | | **Definitely on purpose** | | | | | | | | | | **Possibly on purpose** | | | | | | | | **Possibly an accident** | | | | | | | | **Definitely an accident** | | | | | | |
| **6** | **One of your classmates tried to make your friends exclude you.** | | | | | | | | | | | | | | | | | | | | | | | | | | | | | | | | | | | |
|  | **a.** | | | What would you do (circle one)? | | | | | | | | | | | | | | | | | | | | | | | |  | | | | | |  | | |
|  | | | | **Ignore it** | | | | | | | | **Wait and get even later** | | | | | | | | **Discuss it together and try to solve the problem** | | | | | | **Ask a teacher or other adult for help** | | | **Yell, curse, or call the person names** | | | | | | **Push, hit, or kick the person** | |
|  | **b.** | | | The behavior of your classmate was… | | | | | | | | | | | | | | | | | | | | | | | |  | | | | | |  | | |
|  |  | | | **Definitely on purpose** | | | | | | | | | | **Possibly on purpose** | | | | | | | | **Possibly an accident** | | | | | | | | **Definitely an accident** | | | | | | |
| **7** | **During an argument a classmate swore at you.** | | | | | | | | | | | | | | | | | | | | | | | | | | | | | | | | | | | |
|  | **a.** | | | | What would you do (circle one)? | | | | | | | | | | | | | | | | | | | | | | |  | | | | | |  | | |
|  | | | | | **Ignore it** | | | | | | **Wait and get even later** | | | | | | | **Discuss it together and try to solve the problem** | | | | | | | | **Ask a teacher or other adult for help** | | | **Yell, curse, or call the person names** | | | | | | **Push, hit, or kick the person** | |
|  | **b.** | | | | The behavior of the classmate was… | | | | | | | | | | | | | | | | | | | | | | |  | | | | | |  | | |
|  |  | | | | **Definitely on purpose** | | | | | | | | | | **Possibly on purpose** | | | | | | | | **Possibly an accident** | | | | | | | **Definitely an accident** | | | | | | |
| **8** | **You learned that another student was spreading mean rumors about you.** | | | | | | | | | | | | | | | | | | | | | | | | | | | | | | | | | | | |
|  | **a.** | | | | | | What would you do (circle one)? | | | | | | | | | | | | | | | | | | | | |  | | | | | |  | | |
|  | | | | | | | **Ignore it** | | | | **Wait and get even later** | | | | | | | **Discuss it together and try to solve the problem** | | | | | | | | **Ask a teacher or other adult for help** | | | **Yell, curse, or call the person names** | | | | | | **Push, hit, or kick the person** | |
|  | **b.** | | | | | | | The behavior of the student was… | | | | | | | | | | | | | | | | | | | |  | | | | | |  | | |
|  |  | | | | | | **Definitely on purpose** | | | | | | | | | **Possibly on purpose** | | | | | | | | **Possibly an accident** | | | | | | | **Definitely an accident** | | | | | |
| **9** | **You overheard your friend talking in a group, you heard your name, and some kids laughed.** | | | | | | | | | | | | | | | | | | | | | | | | | | | | | | | | | | | |
|  | **a.** | | | | | | What would you do (circle one)? | | | | | | | | | | | | | | | | | | | | |  | | | | | |  | | |
|  | | | | | **Ignore it** | | | | | **Wait and get even later** | | | | | | | | | **Discuss it together and try to solve the problem** | | | | | | | **Ask a teacher or other adult for help** | | | **Yell, curse, or call the person names** | | | | | | **Push, hit, or kick the person** | |
|  | **b.** | | | | | The behavior of your friend was… | | | | | | | | | | | | | | | | | | | | | |  | | | | | |  | | |
|  |  | | | | | | **Definitely on purpose** | | | | | | | | | **Possibly on purpose** | | | | | | | | **Possibly an accident** | | | | | | | **Definitely an accident** | | | | | |
| **10** | **You told some personal information to a friend and learned the next day that others knew your secret.** | | | | | | | | | | | | | | | | | | | | | | | | | | | | | | | | | | | |
|  | **a.** | | | | | | What would you do (circle one)? | | | | | | | | | | | | | | | | | | | | |  | | | | | |  | | |
|  | | | | | | | **Ignore it** | | | **Wait and get even later** | | | | | | | | | **Discuss it together and try to solve the problem** | | | | | | | **Ask a teacher or other adult for help** | | | **Yell, curse, or call the person names** | | | | | | **Push, hit, or kick the person** | |
|  | **b.** | | | | | | | The behavior of your friend was… | | | | | | | | | | | | | | | | | | | |  | | | | | |  | | |
|  |  | | | | | | **Definitely on purpose** | | | | | | | | | **Possibly on purpose** | | | | | | | | **Possibly an accident** | | | | | | | **Definitely an accident** | | | | | |
| **11** | **In gym a student threw a ball, which hit you hard on the head.** | | | | | | | | | | | | | | | | | | | | | | | | | | | | | | | | | | | |
|  | **a.** | | | | | | What would you do (circle one)? | | | | | | | | | | | | | | | | | | | |  | | | | | |  | | | |
|  | | | | | | | **Ignore it** | | | **Wait and get even later** | | | | | | | | | **Discuss it together and try to solve the problem** | | | | | | | **Ask a teacher or other adult for help** | | | **Yell, curse, or call the person names** | | | | | | **Push, hit, or kick the person** | |
|  | **b.** | | | | | | | The behavior of the student was… | | | | | | | | | | | | | | | | | | |  | | | | | |  | | | |
|  |  | | | | | | **Definitely on purpose** | | | | | | | | | **Possibly on purpose** | | | | | | | | **Possibly an accident** | | | | | | | **Definitely an accident** | | | | | |
| **12** | **You were standing in line for lunch and another student trying to cut into the line in front of you elbowed you in the ribs.** | | | | | | | | | | | | | | | | | | | | | | | | | | | | | | | | | | | |
|  | **a.** | | | | | | What would you do (circle one)? | | | | | | | | | | | | | | | | | | | |  | | | | | |  | | | |
|  | | | | | | | **Ignore it** | | | **Wait and get even later** | | | | | | | | | **Discuss it together and try to solve the problem** | | | | | | | **Ask a teacher or other adult for help** | | | **Yell, curse, or call the person names** | | | | | | **Push, hit, or kick the person** | |
|  | **b.** | | | | | | | The behavior of the student was… | | | | | | | | | | | | | | | | | | |  | | | | | |  | | | |
|  |  | | | | | | **Definitely on purpose** | | | | | | | | | **Possibly on purpose** | | | | | | | | **Possibly an accident** | | | | | | | **Definitely an accident** | | | | | |
